# Knowledge landscape of macrophage research in liver fibrosis: a bibliometric review of the literature from WoSCC

**DOI:** 10.3389/fphar.2025.1571879

**Published:** 2025-05-08

**Authors:** Yanbo Li, Zhengmin Cao, Yanping Lu, Chao Lei, Wenliang Lyu

**Affiliations:** ^1^ Department of Infectious Diseases, Guang’anmen Hospital, China Academy of Traditional Chinese Medicine, Beijing, China; ^2^ Shenzhen Bao’an Chinese Medicine Hospital, Guangzhou University of Chinese Medicine, Shenzhen, China

**Keywords:** liver fibrosis, macrophages, bibliometrics, visualization, pharmacological mechanism

## Abstract

Recent insights into the immune response in fibrosis have provided valuable perspectives for the treatment of liver fibrosis. Macrophages, as the most abundant immune cells in the liver, are key drivers of liver fibrosis. They are extensively involved in tissue damage, chronic inflammation, and the progression and regression of liver fibrosis. This study aims to conduct a bibliometric analysis and literature review on the mechanisms by which macrophages contribute to liver fibrosis. Specifically, we analyzed a bibliometric dataset comprising 1,312 papers from 59 countries, 1,872 institutions, and 9,784 authors. Keyword co-occurrence analysis identified key research hotspots, including the role of macrophage subtypes in obesity-related metabolic disorders, the crosstalk between macrophages and hepatic stellate cells through mechanoimmunology, emerging strategies for immune modulation targeting macrophages to promote fibrosis regression and liver regeneration, and new discoveries regarding macrophage crosstalk with other immune cells. In conclusion, this study provides a visual analysis of the current research landscape, hotspots, and trends in the field of macrophages and liver fibrosis, and discusses future directions for further exploration in this area.

## 1 Introduction

Liver fibrosis is a chronic liver disease caused by pathological factors such as viral infections, non-alcoholic fatty liver disease, or autoimmune diseases (Bataller and Brenner, 2005). The current view holds that the occurrence of liver fibrosis is caused by a series of dynamic cascading reactions triggered by persistent and excessive activation of inflammatory responses in the liver. Uncontrolled chronic inflammation is the main driving force behind the development of liver fibrosis. It transforms the normally self-limiting tissue repair process into a sustained malignant cycle, leading to the activation of hepatic stellate cells (HSCs), accumulation of extracellular matrix (ECM), scarring, and further causing liver hypoperfusion and morphological changes (Kisseleva and Brenner, 2021). The gradual accumulation of fibrotic tissue can ultimately lead to cirrhosis, and may even progress to life-threatening hepatocellular carcinoma (HCC). Epidemiological data show that approximately 2 million people die from liver disease worldwide each year, with around 1 million deaths attributed to cirrhosis and its complications (Asrani et al., 2019). Therefore, inhibiting the progression of liver fibrosis is a key goal in the treatment of chronic liver diseases.

When inflammation occurs in the liver, damaged hepatocytes trigger activation of endothelial cells and recruitment of immune cells. Hepatic macrophages include subtypes such as intrahepatic resident Kupffer cells (KCs) and monocyte-derived macrophages (in this paper, we use the bibliometric convention of using the term “macrophages” to refer to the entire spectrum of the population, and will specify the origin of subtypes when referring to the functional differences). They play an important role in tissue damage, chronic inflammation, the progression of liver fibrosis, and its resolution (Forbes and Rosenthal, 2014). New insights into macrophages have provided unprecedented opportunities for understanding and treating liver fibrosis. Numerous high-quality reviews have already summarized the mechanisms and therapeutic approaches involving macrophages in liver fibrotic diseases (Tacke, 2017; Krenkel and Tacke, 2017; Cheng et al., 2021). However, there is a lack of research that focuses on the current status and future directions of macrophage-related liver fibrosis based on scientific evidence, rather than subjective understanding.

Aiming to provide researchers with an overview of the field of macrophage and liver fibrosis research based on objective evidence, this study visualized and analyzed 1,312 articles from the Web of Science database to explore the current state of research, research hotspots, and trends. New knowledge and future research directions in the field of macrophages and hepatic fibrosis are discussed around keyword clustering analysis at the end of the articles.

## 2 Method

### 2.1 Data sources and search strategy

**FIGURE 1:**
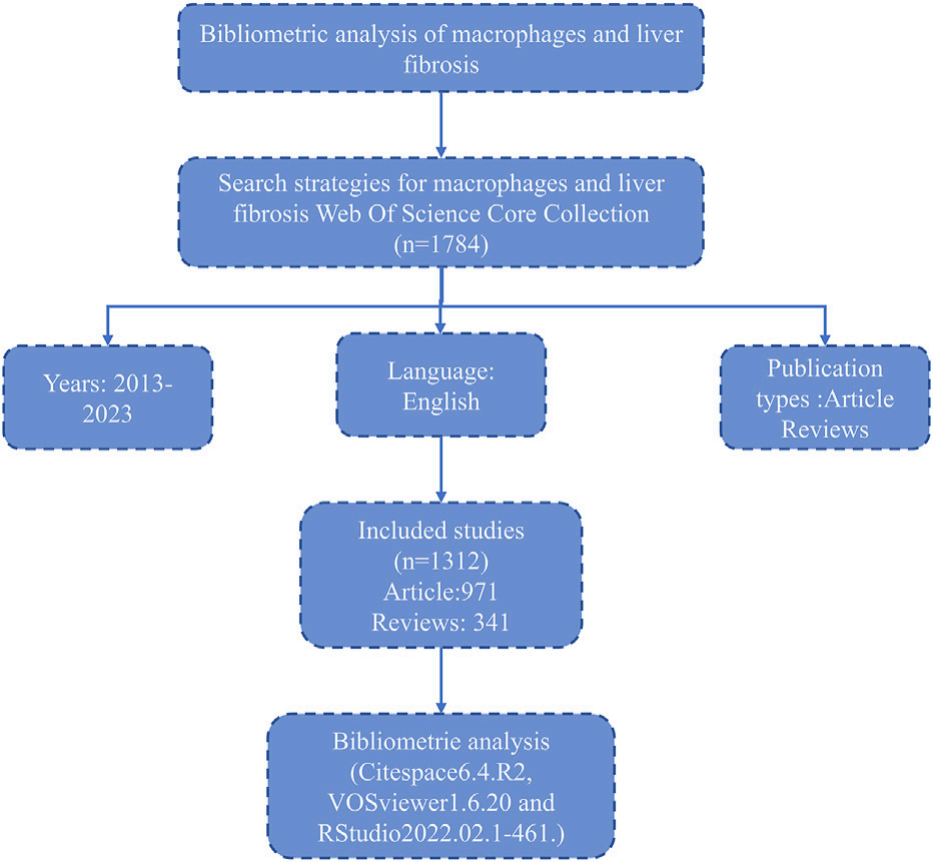
Flowchart for bibliometrics analysis.

Literature from the period 2013-01-01 to 2023-12-31 was downloaded from the Web of Science database for this study. The search strategy used was: (((TS=(macrophage OR macrophages OR “kupffer cell” OR “kupffer cells” OR “kupffer-cell” OR “kupffer-cells”)) AND TS=(“liver fibrosis” OR “hepatic fibrosis” OR “hepatofibrosis”)) AND LA=(English)) AND DOP=(2013-01-01/2023-12-31) Article only. The search terms are selected manually. To ensure the accurate interpretation of the results, only articles and reviews in English were included. A review team consisting of three members independently screened the titles and abstracts of all potentially eligible studies. When disagreements arise, submit to a third-party researcher experienced in the field for adjudication. The complete retrieval process is shown in Figure 1. Ultimately, a total of 1,312 articles were included in the study.

### 2.2 Data processing and visualization

We used VOSviewer1.6.20 and the Bibliometrix package in RStudio2022.02.1-461 for the visualization of collaborations between countries, institutions, and authors, as well as for co-citation analysis of articles and co-occurrence analysis of keywords. Citespace6.4.R2 was used for detecting keyword and citation bursts. To eliminate redundant entries, synonymous expressions were manually normalized. For example, liver fibrosis was standardized as hepatic fibrosis, Hepatic stellate cell as Hepatic stellate cells, liver cancer as Hepatocellular carcinoma.

## 3 Result

### 3.1 Annual publication and citation analysis

**FIGURE 2:**
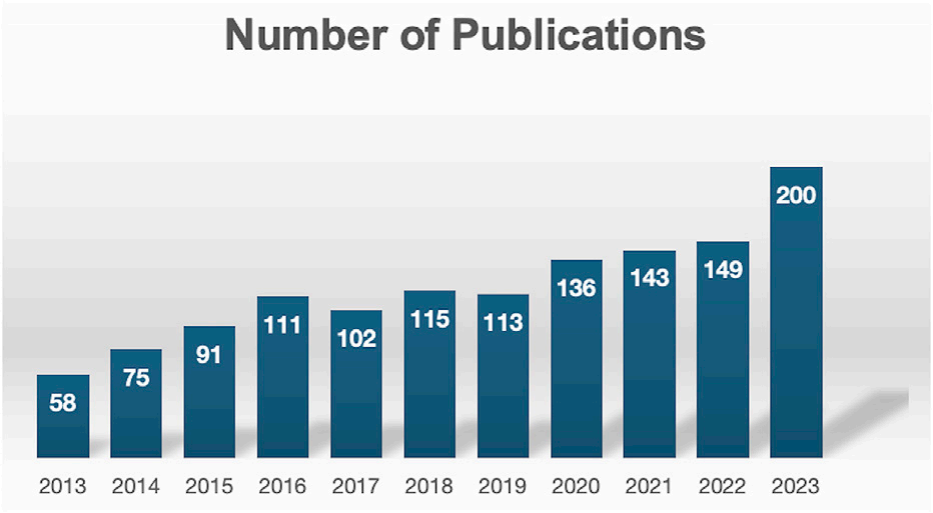
Global publication output on macrophage in liver fibrosis from 2013 to 2023.

This study included 1,312 papers from 59 countries, 1,872 institutions, and 9,784 authors. Figure 2 shows the annual number of publications from 2013 to 2023. Overall, the number of annual publications steadily and rapidly increased. From 2013 to 2015, the number of related articles grew significantly. The annual publication volume increased from 58 in 2013 to 91 in 2015, a growth rate of approximately 56.9%. From 2016 to 2022, the annual growth rate slowed, with the number of publications remaining above 100 each year. Between 2022 and 2023, the annual publication volume sharply increased from 149 to 200 papers, a rise of 51 papers, corresponding to an increase of approximately 34.2%, reflecting a rapid surge in annual publication numbers. In summary, these findings indicate sustained and focused attention from researchers in the field of macrophages and liver fibrosis.

### 3.2 Analysis of the highest publication countries/regions

**FIGURE 3:**
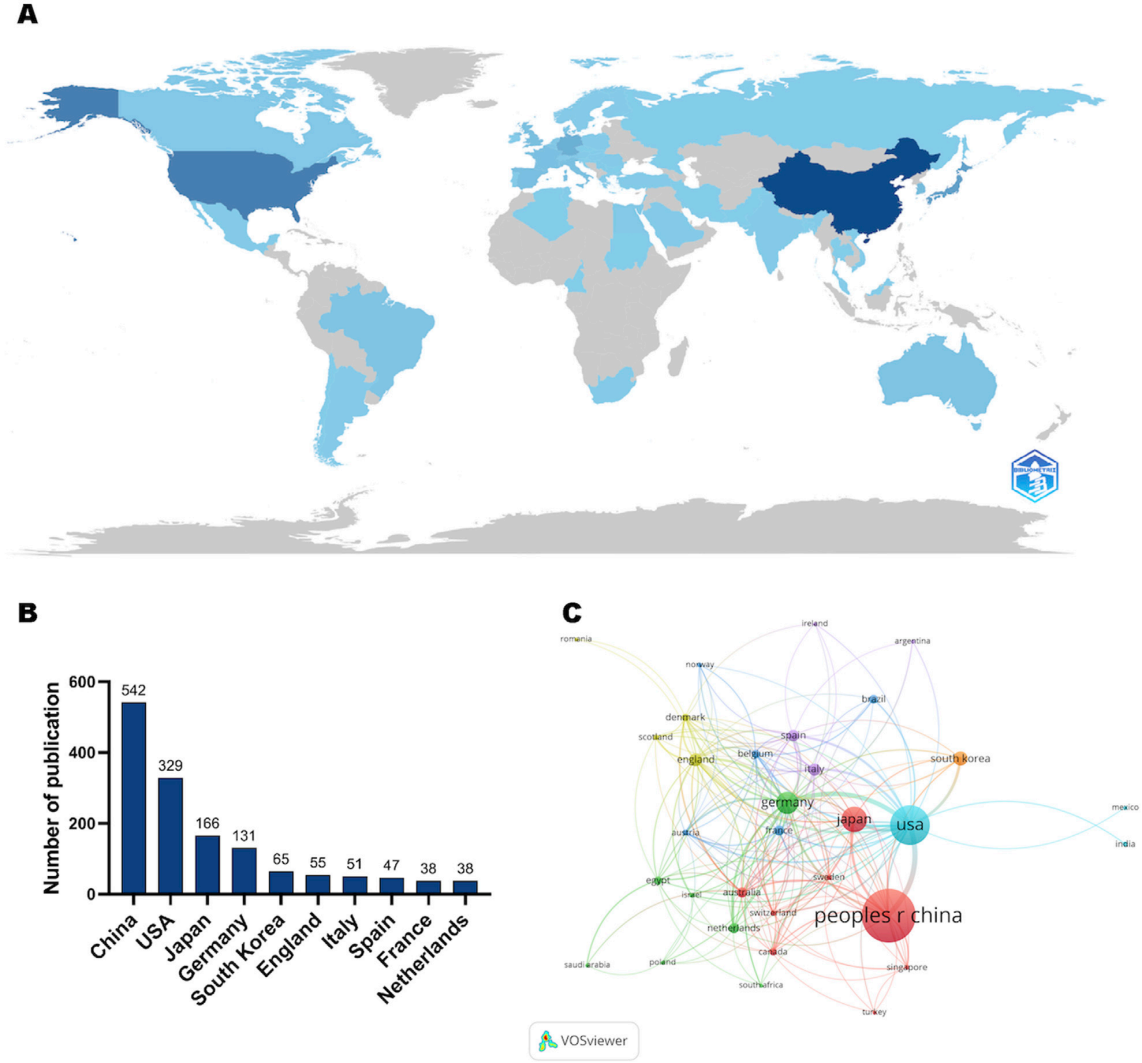
**(A)** Geographic distribution map based on the total volume of publications for different countries/areas. **(B)** The top ten countries/regions in total number of publications. **(C)** Visualization map of country/region citation networks generated using the VOS browser. The thickness of the lines reflects the strength of the citations.

According to the bibliometric analysis, most papers related to macrophages and liver fibrosis were published in countries such as those in Asia and North America (Figures 3A,B). Among them, China published the most papers (41.31%, 542 papers), followed by the United States (25.07%, 329 papers) and Japan (12.65%, 166 papers) (Table 1). Figure 3C shows the international collaboration between countries/regions, revealing that the major connections are concentrated between China, Japan, the United States, Germany, and other countries.

**TABLE 1:** Top 10 countries/institutions with the highest number of publications.

Rank	Country	Documents	Citations	Total link strength
1	China	542	13,133	137
2	United States	329	16,782	308
3	Japan	166	4,812	64
4	Germany	131	8,823	211
5	South Korea	65	1782	36
6	England	55	3,944	106
7	Italy	51	2,813	60
8	Spain	47	1702	69
9	France	38	2,286	46
10	Netherlands	38	1,479	41

### 3.3 Analysis of the highest publication institutions

**FIGURE 4:**
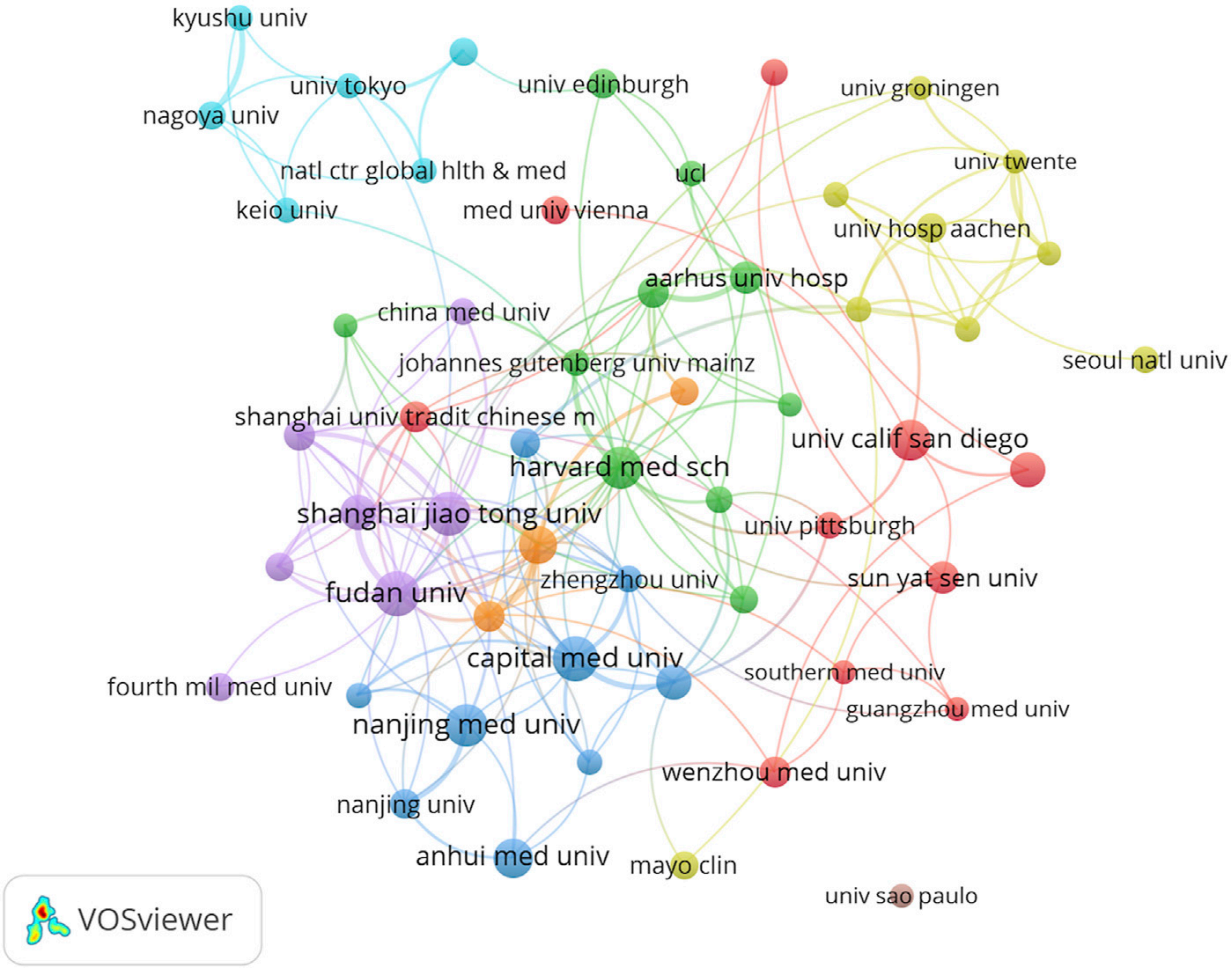
Visualization of institutional collaboration networks.

A total of 1,872 institutions contributed to research in the field of macrophages and liver fibrosis. Table 2 lists the top ten institutions by publication volume. Both the United States and China have a large number of institutions involved in scientific research. The majority of papers come from institutions such as Capital Medical University, Fudan University, Shanghai Jiaotong University, Nanjing Medical University, and Harvard Medical School. The collaboration between institutions is shown in Figure 4, where Fudan University and Shanghai Jiaotong University represent one group (depicted in purple), while Capital Medical University, Nanjing Medical University, and Nanjing University represent another group (depicted in blue). Currently, collaboration between research institutions is mainly concentrated within regions of the same country, with limited international exchange and cooperation.

**TABLE 2:** Top 10 organizations with the highest number of publications.

Rank	Organization	Documents	Citations	Total link strength
1	Capital Medical University	34	932	29
2	Fudan University	34	1,276	39
3	Shanghai Jiaotong University	32	959	39
4	Nanjing Medical University	30	676	15
5	Harvard Medical School	29	1,071	29
6	University of California, San Diego	28	2,516	8
7	Anhui Medical University	25	712	6
8	Chinese Academy of Sciences	24	1,172	43
9	Sichuan University	21	464	17
10	Huazhong University of Science and Technology	20	558	4

### 3.4 Journal and co-citation analysis

**FIGURE 5:**
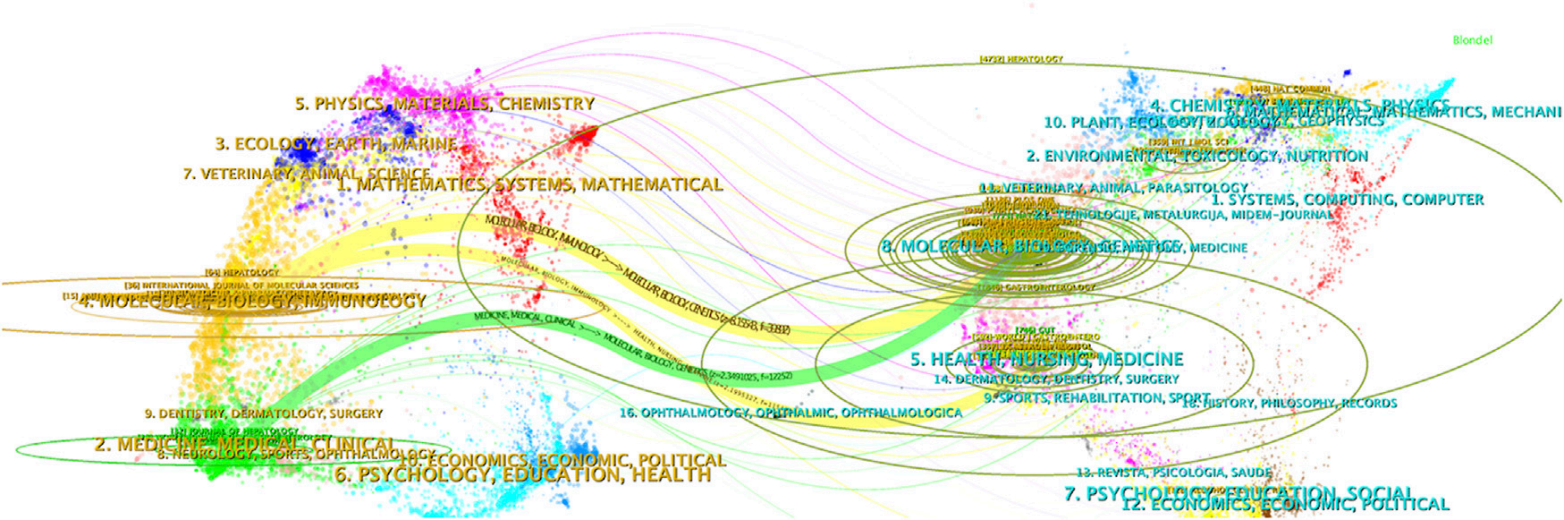
Dual-map overlay Dual-map overlay results show the distribution, citation trajectory of papers between disciplines. On the left is the sizing map, on the right is the cited map, and the curves are citation links, the width of which corresponds to the intensity of the cross-citation.

A total of 1,312 papers were published in 434 journals. Table 3 lists the top 10 journals by publication volume. The top three journals are Hepatology (n = 64), PLOS One (n = 51), and Scientific Reports (n = 40). Among them, Journal of Hepatology has the highest impact factor (IF) of 26.8. Table 4 shows the top 10 journals by citation volume, with the most-cited journals being Hepatology (IF = 13), Journal of Hepatology (IF = 26.8), and Gastroenterology (IF = 26.3). Figure 5 “Dual-map overlay of journals in which research was published” illustrates the distribution of journals and citation themes. The results indicate that articles published in Molecular/Biology/Immunology and Medicine/Medical/Clinical journals tend to cite papers from journals in Molecular/Biology/Genetics.

**TABLE 3:** Top 10 journals with the highest number of publications.

Rank	Journal	Count	IF	JCR
1	Hepatology	64	13	Q1
2	Plos One	51	2.9	Q3
3	Scientific Reports	40	3.8	Q2
4	International Journal of Molecular Sciences	36	4.9	Q2
5	Frontiers in immunology	33	5.7	Q2
6	Journal of Hepatology	32	26.8	Q1
7	Biomedicine & Pharmacotherapy	20	6.9	Q2
8	International Immunopharmacology	19	4.8	Q2
9	World Journal of Gastroenterology	18	4.3	Q3
10	American Journal of Physiology- gastrointestinal and Liver Physiology	15	3.9	Q3

**TABLE 4:** Top 10 most cited journals.

Rank	Co-cited journal	Citations	IF	JCR
1	Hepatology	4,737	13	Q1
2	Journal of Hepatology	2,806	26.8	Q1
3	Gastroenterology	1846	26.3	Q1
4	Journal of Clinical Investigation	1,468	13.3	Q1
5	Plos One	1,188	2.9	Q3
6	Journal of Immunology	1,092	3.6	Q3
7	Journal of Biological Chemistry	1,047	4.0	Q2
8	Proceedings of the National Academy of Sciences	930	9.4	Q1
9	Nature	773	50.5	Q1
10	Gut	748	23.1	Q1

### 3.5 Analysis of authors and cited authors

**FIGURE 6:**
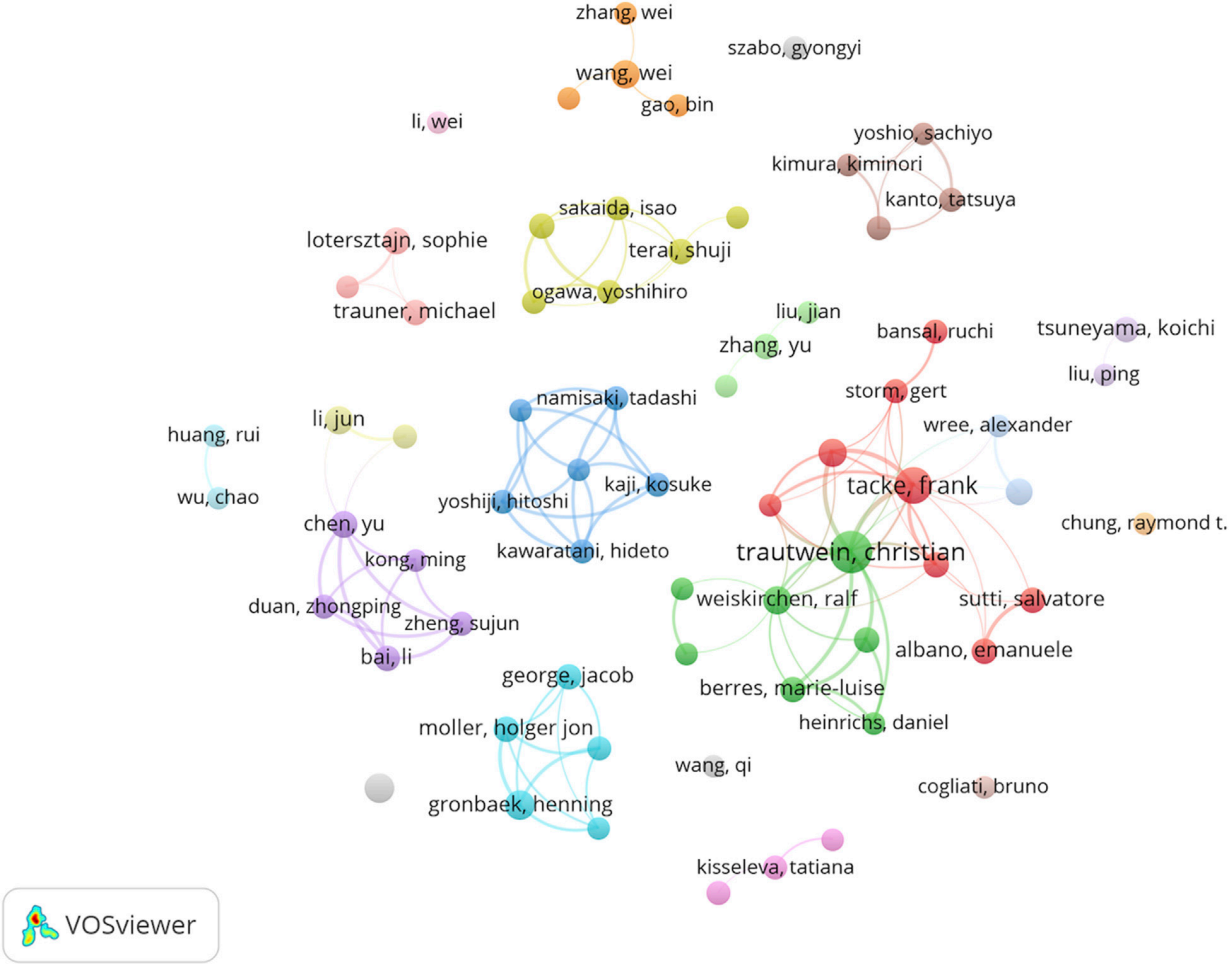
Authors’ collaboration network.

A total of 9,784 authors were identified as contributors to the field. Table 5 summarizes the top 10 authors by publication count and co-citation count. Among the high-output authors, the most published papers were by Trautwein Christian (24 papers) and Tacke Frank (18 papers). Notably, although Friedman SL only published 9 articles, his works were cited 469 times, ranking first. Following him were Seki E (n = 313) and Tacke F (n = 304). Figure 6 displays the author collaboration network, showing that nine major collaborative teams have made significant contributions to the field of macrophages and liver fibrosis. However, the collaboration between these teams is not very close, indicating that widespread cooperation will be essential for the future development of the field.

**TABLE 5:** Top 10 authors and co-cited authors.

Rank	Authors	Count
1	Trautwein Christian	24
2	Tacke Frank	18
3	Gronbaek Henning	11
4	Schuppan Detlef	11
5	Li Jun	10
6	Luedde Tom	10
7	Wang Wei	10
8	Weiskirchen Ralf	10
9	Chen Yu	9
10	Feldstein Ariel E	9

Rank	Co-cited Authors	Count
1	Friedman SL	469
2	Seki E	313
3	Tacke F	304
4	Wynn Ta	276
5	Ramachandran P	240
6	Bataller R	207
7	Kisseleva T	185
8	Duffield JS	161
9	Schuppan D	154
10	Krenkel O	151

### 3.6 Analysis of highly cited references

**FIGURE 7:**
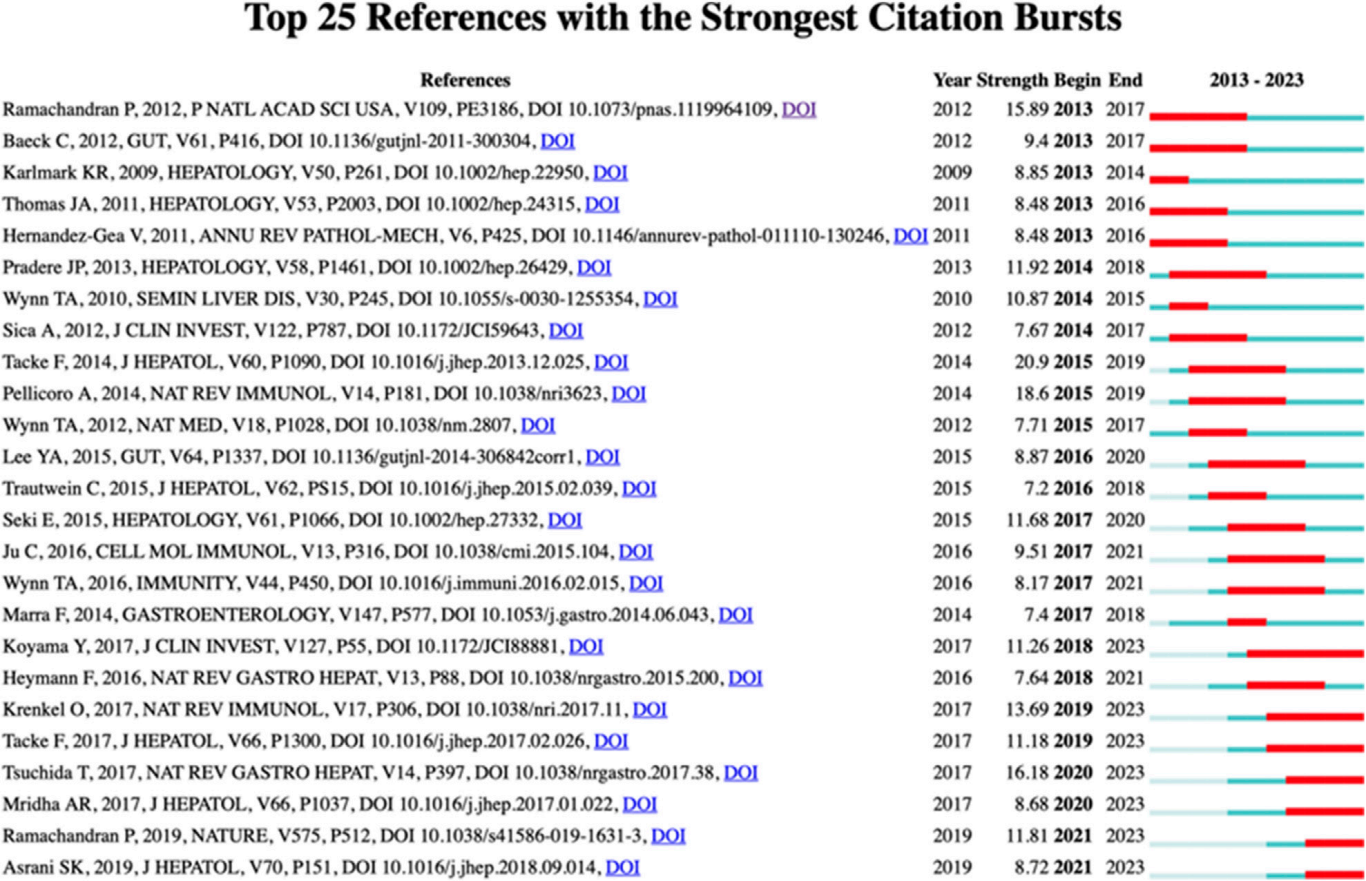
Visualization map of top 25 references with the strongest citation bursts.Literature information on the left, citation intensity, citation burst time on the right.

Figure 7 presents the 25 most highly cited references in the field of macrophages and liver fibrosis. Notably, the majority of these references originate from top-tier academic journals, such as Hepatology, Gut, and Immunity. Most citation peaks occurred after 2009. The most frequently cited paper is titled Macrophage Heterogeneity in Liver Injury and Fibrosis, which comprehensively reviews the complex roles of macrophage subpopulations in liver homeostasis, inflammatory responses, and liver injury and fibrosis. It particularly highlights the key roles of macrophages derived from different cellular origins in promoting injury repair and resolution. Of special note, approximately 15 of these 25 highly cited references are review articles, underscoring the significant role of reviews in academic communication and knowledge integration.

### 3.7 Keyword analysis

**FIGURE 8:**
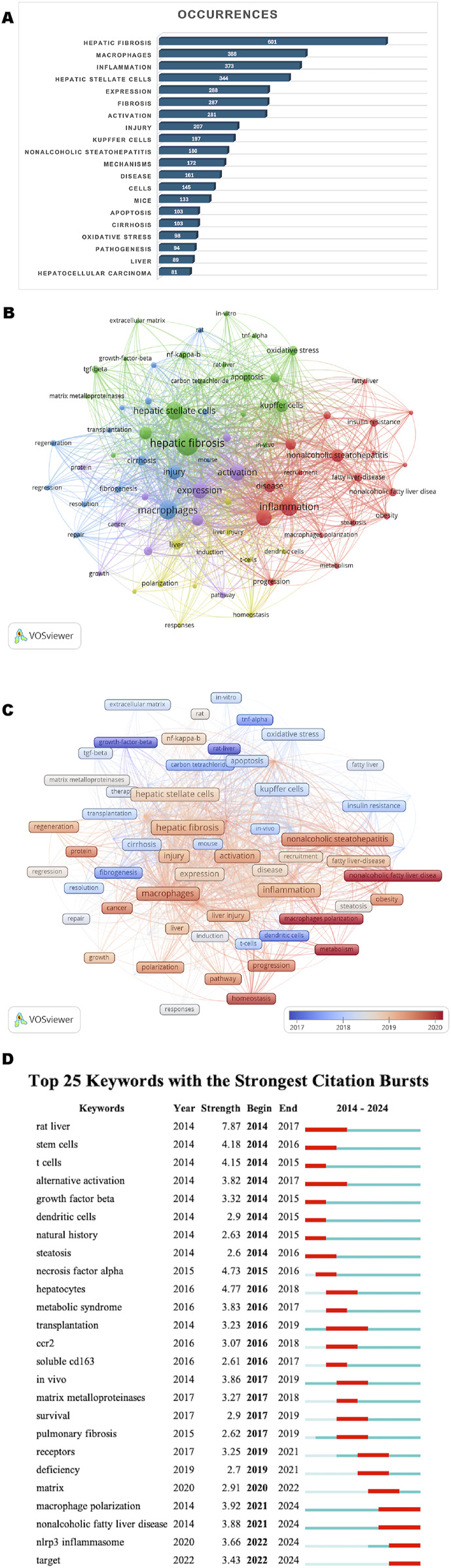
**(A)** The top 20 keywords ranked by frequency of occurrence. **(B)** Keyword co-occurrence diagram. The same color represents a class of keywords and a total of 5 clusters were obtained. **(C)** Timeline diagrams of keywords. Keywords from earlier periods are marked in blue and purple, while newer keywords are marked in orange, with the most recent keywords highlighted in red. **(D)** Top 25 keywords with the strong citation burst.

Keywords reflect the core topics and research hotspots in the field of macrophages and liver fibrosis. In this study, a total of 4,750 keywords were collected, and the top 74 most frequent keywords, each appearing at least 25 times, were extracted and clustered. Figure 8A displays the top 20 keywords ranked by frequency of occurrence. In addition to *hepatic fibrosis* (601) and *macrophages* (388), other frequently occurring keywords in this study include inflammation (373), *hepatic stellate cells* (344), expression (288), *fibrosis* (278), activation (281), *injury* (207), *Kupffer cells* (197), and *nonalcoholic steatohepatitis* (180), indicating that these are the focal points of the research in this area.

Using VOSviewer, a keyword cluster network map (Figure 8B) was generated, dividing the keywords into five clusters, each representing a distinct research direction in the field of macrophages and liver fibrosis. The five clusters, containing 19, 19, 15, 11, and 10 keywords respectively, highlight the key areas of research. Cluster 1 (Red): This group focuses on the mechanisms of macrophage involvement in non-alcoholic fatty liver disease (NAFLD), including keywords such as adipose tissue, inflammation, insulin resistance, macrophage polarization, metabolism, obesity, progression, and recruitment. Cluster 2 (Green): This cluster is concerned with the mechanisms of macrophages in promoting fibrosis, including terms like apoptosis, extracellular matrix, hepatic stellate cells, matrix metalloproteinase, nuclear factor-kappa B (NF-κB), oxidative stress, transforming growth factor-β (TGF-β), and tumor necrosis factor alpha (TNF-α). Cluster 3 (Blue): This group focuses on the role of macrophages in injury repair responses, including differentiation, regeneration, regression, repair, resolution, therapy, and transplantation. Cluster 4 (Yellow): This cluster examines the interactions of macrophages with other immune cells, including dendritic cells, homeostasis, induction, monocytes, receptor, responses, and T-cells. Cluster 5 (Purple): This group centers on the role of macrophages in liver cancer (Hepatocellular carcinoma), with keywords such as activation, cancer, expression, growth, hepatocellular carcinoma, inhibition, and proliferation. These five clusters represent the primary research directions within the field of macrophages and liver fibrosis.

Figure 8C presents a temporal overlap visualization of keywords. The results indicate that early research on macrophages and liver fibrosis focused on topics such as apoptosis, oxidative stress, KCs, resolution, matrix metalloproteinases, and T-cells, suggesting that early research hotspots revolved around the complex mechanisms of macrophage involvement in liver fibrosis (green, blue, and yellow clusters). More recent research has concentrated on keywords like macrophage polarization, NAFLD, homeostasis, and metabolism, suggesting that current research hotspots are likely related to macrophage involvement in NAFLD-related liver fibrosis (red cluster). This analysis highlights how research trends have evolved over time, with a shift towards newer topics and areas of focus.

Figure 8D reports the top 25 keywords with the strongest citation bursts related to macrophages and liver fibrosis. As shown in the figure, the blue line represents the timeline, and the red segments on the blue timeline indicate citation bursts, marking the start year, end year, and duration of the burst. Notably, “rat liver” (7.87) exhibits the strongest citation burst, followed by “hepatocytes” (4.77) and “necrosis factor alpha” (4.73). Based on the starting time of appearance, terms such as “rat liver,” “stem cells,” “T cells,” “alternative activation,” and “growth factor beta” emerged earlier and were the focus of early attention. “Matrix,” “macrophage polarization,” “nonalcoholic fatty liver disease,” “NLRP3 inflammasome,” and “target” represent the current research frontiers in macrophages and liver fibrosis and are now in the burst phase. This suggests that future research may focus on targeted therapies, inflammasomes, extracellular matrix, and the regulation of macrophage polarization phenotypes in relation to NAFLD.

## 4 Discussion

### 4.1 Global trends on macrophages and liver fibrosis

To the best of our knowledge, this is the first bibliometric study on liver fibrosis and macrophages that reviews research trends in the field. Our findings reveal significant findings, research interests, and frontiers in the biomechanics of liver fibrosis. We analyzed 1,312 papers authored by 9,784 researchers and found a rapid increase in publications over the past decade, especially after 2018. This indicates a growing interest among researchers in the field of macrophages and liver fibrosis. We believe that the following factors have contributed to the rapid increase in the number of launches: (Bataller and Brenner, 2005): breakthroughs in technological bottlenecks, the maturation of single-cell sequencing technology has enabled researchers to finely resolve the heterogeneous characteristics of liver macrophages for the first time, and innovations in spatial transcriptome technology have enabled researchers to dynamically tracer macrophages in a three-dimensional microenvironment; (Kisseleva and Brenner, 2021); with the rise in the global prevalence of NAFLD, the anti-hepatofibrotic therapeutic strategy is in demand has increased; (Asrani et al., 2019); macrophages are involved in the entire process of innate immune response and tissue repair, and their complex mechanism of action has attracted the interest of researchers.

The results of the institutional analysis show (Table 2) that Chinese institutions dominate in terms of publication volume (8/10 former institutions), which hints at the level of interest in this issue among Chinese researchers, and the collaboration network (Figure 2) demonstrates extensive collaboration among domestic institutions (Shanghai Jiao Tong-Fudan-Capital Medical Axis). However, the collaboration between domestic and foreign institutions is limited, which may limit the recognition of the results of these institutions to some extent. The citation rate of articles reflects this issue to some extent, with Harvard Medical School, ranked fifth, maintaining a citation/publication ratio of 36.9 (1071/29), almost three times that of Fudan University (32.7/1276/39). Extensive East-West collaborations (East Asia-Europe/USA) as well as academic institution-pharmaceutical company collaborations are necessary to help increase the reach of results and facilitate translation.

Bibliometric analyses revealed the number of journal publications and citations in macrophage-associated liver fibrosis research (Tables 3, 4). Hepatology (IF = 13, Q1) led the way in terms of publications (You et al., 2008) and citations (4,737). The results in Figure 5 are suggestive of the widespread pattern of interdisciplinary collaboration in research in this field, encompassing the disciplines of molecular biology, immunology, and genetics. The parallel appearance of clinical and molecular biology and immunology specialty journals reflects the coexistence of dual directions of development in the field: mechanism exploration and therapeutic development. Innovation stems from multidisciplinary integration, and interdisciplinary expertise will provide novel perspectives and knowledge for research in the field.

### 4.2 Hot spots, frontiers and teamwork in the field of macrophages and liver fibrosis

The analysis of the cited outbreak literature (Figure 6) represents, to some extent, the work of greatest interest to researchers in the field of macrophages and liver fibrosis during the calendar year, and to some extent reflects the development of the field over the years. In 2013, there was a general focus on the classification of macrophage subpopulations and their specific roles in the process of liver fibrosis. For example, the origin and pro-restorative role of CD11B^hi^ F4/80^int^ Ly6C^lo^ macrophages (Ramachandran et al., 2012) and the pro-inflammatory, pro-fibrotic role of CD11b^+^ F4/80^+^ Ly6C^hi^ monocytes (Karlmark et al., 2009). In addition to mechanism exploration, anti-hepatic fibrosis therapeutic strategies targeting macrophages have also been a hot research topic, and the discovery of CC chemokine ligand 2 (CCL2) antagonists (Baeck et al., 2012) and bone marrow-derived macrophages (Thomas et al., 2011) have attracted extensive attention in the field. As research progressed, specific mechanisms of macrophage pro-fibrosis were revealed, with macrophages activating hepatic stellate cells and promoting liver fibrosis in an NF-κB-dependent manner (Pradere et al., 2013). Subsequently, the highly cited literature between 2014 and 2023 was dominated by review articles, suggesting that the field of macrophage and liver fibrosis research has entered a stage of in-depth analysis, and the complex regulatory network of macrophages is gradually clarified, and a large number of breakthroughs need to be systematically summarized and sorted out.

Interesting information can be obtained by analyzing Figure 7 in conjunction with the author collaboration network (Figure 5), where Trautwein Christian and Tacke Frank are the first and second authors with the highest number of publications in the field. The collaboration between the two in the field of liver disease is a landmark scientific partnership in the field. Their original studies published in 2009 and 2012 identified pro-inflammatory Ly6C monocytes that enter the damaged liver in a CCL2/cc chemokine receptor 2 (CCR2) axis-dependent manner and demonstrated that targeting CCL2 inhibits macrophage-driven inflammation and angiogenesis (Karlmark et al., 2009; Baeck et al., 2012). These results were included in high-impact journals such as Gut, Hepatology, and received an explosion of citations in the field from 2013 to 2017. They have co-authored several influential papers in the liver disease industry, covering topics such as migratory differentiation mechanisms of macrophages (Heymann et al., 2012) and drug delivery systems targeting macrophages (Ergen et al., 2019), which have profoundly advanced the field from multiple perspectives, including mechanism exploration and drug development. Their collaboration has also led to the participation of other researchers, such as Salvatore Sutti, Gert Storm, etc., laying the foundation of multiple team networks.

The collaborative efforts of prolific authors such as Kong ming and chen yu have likewise improved the field’s understanding of the complex relationship between macrophages and liver fibrosis. Their findings revealed the role of M2-type macrophages in acute-on-chronic liver failure in regulating the necroptosis-S100A9-necroinflammation axis, pyroptosis of synergistic hepatoprotective mechanisms (Bai et al., 2022; Bai et al., 2021). Their collaboration attracted more collaborators to join them. Kong ming, in collaboration with Xiaowei Liu and Chunjing Guo, combined nanocomposite microneedle technology with astragalus polysaccharide to construct the first functionalized drug delivery system targeting skin macrophages (Liu et al., 2024). This breakthrough result was made possible by the collaboration between the pharmacy, nanotechnology and basic immunology teams.

Overall, the collaborative efforts among researchers led to key discoveries in the field of macrophages and liver fibrosis, improving our understanding of the complex relationship between macrophages and liver fibrosis while paving the way for the development of new therapeutic strategies to combat liver disease. The results of the collaboration between top teams and authors are widely discussed, and to a certain extent, they even lead the hotspots and frontiers of the field. In addition, the interdisciplinary collaboration network facilitates the effective utilization of research resources and promotes further research and innovation in the research field.

### 4.3 Discussion of research hotspots based on keyword analysis

The clustering analysis and citation explosion analysis of keywords can represent the research hotspots and directions in the field of macrophage and liver fibrosis to a certain extent (Figures 8B, D), while the timeline analysis (Figure 8C) can help to understand the current hotspots in the field. The combination of the two analyses yielded interesting results (Bataller and Brenner, 2005): The red clusters contain the hot keywords “metabolism” and “NAFLD” in 2020, as well as obesity, etc. Therefore, we believe that the current hotspots of macrophage research in NAFLD are mainly focused on obesity and metabolism, and are discussed in the following (Kisseleva and Brenner, 2021). The key words in the green cluster are mainly related to the pro-fibrotic mechanism of macrophages, in which macrophages and hepatic stellate cells are the “protagonists”, as well as extracellular matrix and matrix metalloproteinases. Since the signaling pathway-mediated crosstalk mechanism between macrophages and hepatic stellate cells has been summarized in several excellent reviews (Matsuda and Seki, 2020), we attempted to explore the new findings of the crosstalk mechanism between macrophages and hepatic stellate cells from the perspective of mechanical and structural changes in the extracellular matrix (Asrani et al., 2019). The yellow cluster focuses on the interaction between macrophages and other immune cells, and also contains the hot keyword “homeostasis”, so we try to discuss the immune tolerance mechanism of liver immune cells such as macrophages (Forbes and Rosenthal, 2014). Combined with the keywords in the blue cluster, we found that macrophage phenotypic transformation affects the hepatic fibrosis damage repair response, and targeting macrophages to promote hepatic fibrosis regression has become an increasing research hotspot. The keywords in the purple cluster are centered around macrophages and hepatocellular carcinoma, which have limited relevance to the topic of this study, and therefore are not discussed here.

#### 4.3.1 New discoveries on macrophage subtypes involved in obesity-related metabolic disorders

The polarization of hepatic macrophages is a key feature in the development of Non-alcoholic Steatohepatitis (NASH) and is closely associated with disease progression (Alisi et al., 2017). NAFL develops due to long-term consumption of high carbohydrates or fats, leading to lipid accumulation in hepatocytes and hepatocyte death. In this inflammatory state, hepatocytes secrete chemokines, which activate KCs and induce the infiltration of CCR2^+^ Ly6C^+^ monocytes. These monocytes then differentiate into monocyte-derived macrophages (Obstfeld et al., 2010). The role of macrophages in NAFLD extends beyond a simple immune response, involving complex pathological mechanisms such as obesity, insulin resistance, and adipogenesis in NAFLD. Several excellent reviews have already discussed these topics in detail (Alabdulaali et al., 2023; Vonderlin et al., 2023; Barreby et al., 2022).

Obesity is one of the major risk factors for NAFLD, accompanied by the enlargement of adipose tissue, dysfunction of adipocytes, and the exacerbation of insulin resistance (IR). During the progression of obesity, the number of macrophages in adipose tissue (ATM) significantly increases, with their sources including resident macrophages and macrophages derived from recruited monocytes (Gwag et al., 2021). A large body of data suggests that ATM plays a crucial role in regulating insulin sensitivity (Klöting et al., 2010) (Lanthier et al., 2010). Increasing evidence suggests that the classification of macrophages into M1 and M2 phenotypes is overly simplistic, as more macrophage subpopulations and their complex roles are gradually being discovered. Lipid-associated macrophages (LAM) have been identified as a rapidly expanding immune cell subset in mouse adipose tissue, with typical markers including Trem2, Lgals3, and Ctsl. LAMs are primarily found in the crown-like structures (CLS) surrounding enlarged, dying, or dead adipocytes (Grosjean et al., 2021). LAM is generally considered protective, with anti-inflammatory and tissue remodeling functions. It may prevent the exacerbation of metabolic disorders by promoting cell death-prone adipocytes and is associated with lipid metabolism through the lipid receptor Trem2 (Jaitin et al., 2019). Another typical subset is vascular-associated macrophages (VAM), which are closely associated with blood vessels and exhibit a phenotype consistent with M2 polarization. They originate from the resident macrophage population (Silva et al., 2019). However, the regulatory role of VAM in metabolism has not been extensively reported, and indirect effects cannot be ruled out. Single-cell RNA sequencing (scRNA-seq) has provided new insights in exploring the heterogeneity of ATMs. A typical example is the discovery of vascular-associated macrophages (ResVAM) and different metabolically active macrophages (MMacs), which are localized around the adipose tissue vascular system and dispersed throughout the tissue (Boesch et al., 2024). The localization of macrophage subsets’ infiltration provides valuable information that can help us better assess their roles. The aforementioned macrophage subsets are believed to potentially participate in the protective function of the vascular system barrier, which may contribute to maintaining the balance of metabolic homeostasis. Additionally, in both obese human populations and mouse models, a mixed phenotype of pro-inflammatory and metabolic alteration has been identified in the ATM subset, namely, ATF4^hi^PDIA3^hi^ACSL4^hi^CCL2^hi^ inflammatory and metabolically activated macrophages (iMAMs). Among them, PDIA3 is a key effector molecule that transduces metabolic stress signals and macrophage pro-inflammatory reprogramming (Luo et al., 2024). In conclusion, the new discoveries of macrophage subsets provide a fresh perspective for a comprehensive understanding of the role of macrophages in metabolic disorders and insulin resistance.

It is worth noting that while animal models have demonstrated a key role of ATMs in insulin resistance, studies from human populations are relatively scarce, and some have even reported contradictory findings. A clinical cohort-based study indicated that there was no significant correlation between adipose inflammation and the levels of the adipose insulin resistance marker IC50. Moreover, the content of pro-inflammatory ATMs was negatively correlated with insulin resistance in adipose tissue following weight loss (Espinosa De Ycaza et al., 2022). This suggests that animal models may only reflect a partial representation of the disease mechanisms in human NASH and do not fully represent the human condition. Our understanding of human macrophage populations and their relationship with obesity and metabolic disorders still has significant gaps.

#### 4.3.2 A new discovery of the crosstalk mechanism between macrophages and hepatic stellate cells based on mechanobiology of immunology

Hepatic mesenchymal cells, located in the perisinusoidal space between fenestrated hepatic endothelial cells and epithelial hepatocytes, play a central role in liver fibrosis. A substantial body of data indicates that the crosstalk between hepatic stellate cells and macrophages, mediated by cytokines and chemokines, plays a crucial role in the progression of liver fibrosis. Key pathways involved in this crosstalk include the XBP1/STING signaling pathway, (Yu et al., 2019), the ERK-TGFβ1 pathway, (Cai et al., 2020), and the monocyte chemoattractant protein-1/CCR2 axis (Li et al., 2023). These concepts have been extensively discussed in several excellent reviews (Matsuda and Seki, 2020). As liver fibrosis progresses, the mechanical and structural changes in the extracellular matrix play a significant role in influencing cell phenotypes and functions. This role is crucial in the crosstalk between macrophages and hepatic stellate cells, which falls under the domain of mechanobiology. Mechanobiology transcends the limitations of traditional molecular biology research by focusing on how alterations in the mechanical microenvironment affect the immune cells within it. This field encompasses a broad range of mechanical factors in the body, such as extracellular matrix stiffness, fluid shear stress, and cell tension and compression forces, all of which exert comprehensive effects on various immune cells.

Matrix stiffness is determined by the rigidity of the extracellular matrix, which is influenced by its composition, density, spatial orientation, and degree of crosslinking (Chaudhuri et al., 2020). A substantial body of data suggests that increased matrix stiffness is not only a consequence of liver fibrosis but also an active driver of the progression of fibrotic liver diseases (Liu et al., 2021; Lachowski et al., 2019; Kostallari et al., 2022). Macrophages exhibit high plasticity in response to changes in matrix stiffness. *In vitro* models with stiffer substrates show that macrophages stimulated by agents such as LPS and TNF-α produce more inflammatory cytokines, pro-inflammatory mediators, and exhibit enhanced phagocytic function (Patel et al., 2012; Blakney et al., 2012; Previtera and Sengupta, 2015). The soft matrix stiffness in the study ranged from 0.3 to 2 kPa, while the stiff substrates had stiffness values ranging from 130 to 840 kPa. Furthermore, macrophage phenotypic transformation also follows a pattern in response to increased substrate stiffness. Bone marrow-derived macrophages (BMDM) exhibit a pro-inflammatory M1 phenotype on stiff substrates, while they demonstrate an anti-inflammatory M2 phenotype on soft substrates (Mei et al., 2024). However, this conclusion remains somewhat controversial. In a breast cancer tumor mouse model, a stiffer tumor microenvironment was associated with a higher number of M2 macrophages and a significant reduction in M1 macrophages (Taufalele et al., 2023). This result was validated by scRNAseq, flow cytometry, and immunostaining data. A similar phenomenon was observed on substrates with stiffness of 6 kPa, 10 kPa, and 16 kPa, where increased matrix stiffness enhanced M2 polarization of macrophages (Xing et al., 2021). The differences in these findings may be due to variations between *in vivo* and *in vitro* experiments. Additionally, the design of different matrix stiffness gradients could lead to different conclusions. Existing data reveal a general trend: on softer matrices (0.3–16 kPa), macrophages shift towards the anti-inflammatory M2 phenotype; on stiffer matrices (130–840 kPa), macrophages shift towards the pro-inflammatory M1 phenotype. Current research on the mechanisms by which matrix stiffness influences macrophage phenotypic transformation mainly focuses on mechanosensitive signaling pathways, with Piezo1 being the most representative. Piezo1is a non-selective Ca^2+^-permeable ion channel receptor that can be activated by changes in matrix stiffness as well as various mechanical stimuli (Coste et al., 2010; Lin et al., 2019). A large body of data suggests that macrophages cultured on rigid substrates show elevated levels of pro-inflammatory factors and activation of the Piezo1 pathway (Solis et al., 2019).

The activation of HSCs is influenced by increased matrix stiffness, (Sun et al., 2023), a process accompanied by the activation of integrin β1 and the nucleation and activation of Yes-associated protein (YAP) (Chen et al., 2023). YAP and integrin are also mechanosensitive signaling pathways that mediate macrophage phenotypic transformation in response to matrix stiffness (Wang et al., 2022). Therefore, it can be concluded that there exists a complex crosstalk between HSCs and macrophages, and that matrix stiffness can influence macrophage phenotypic switching and promote HSC activation through mechanosensitive signaling pathways. This novel finding provides a new perspective for better understanding the crosstalk between the two.

The impact of fluid shear stress on macrophages is another key and complex research area in mechanobiology. Within the multiscale environment of living organisms, different fluid linkages exist, and the complex fluid behavior forms the flat connections underlying various biological functions (Liu et al., 2022). Therefore, we can reasonably assume that the effect of fluid shear stress on macrophages and hepatic stellate cells may be more direct and rapid. A common understanding is that significantly increased fluid shear stress can induce macrophage activation, accompanied by inflammasome activation and increased cytokine levels. The Piezo1 signaling pathway plays an essential role in this process, with the conformational change of the Piezo1 mechanosensitive ion channel activating NF-kB and NLRP3, resulting in the production of a series of cytokines (Fish and Kulkarni, 2024). However, there is some controversy regarding the effect of shear stress on macrophage phenotypic transformation. In *in vitro* models using 3D microfluidic tissue culture systems to simulate interstitial fluid flow (3 μm/s), macrophages polarized toward the M2 phenotype, as evidenced by the upregulation of M2 markers such as Arg-1, TGF-β, CD206, CD163, CD86, and Transglutaminase 2, along with enhanced migration speed (Li et al., 2018). Conversely, other studies have shown different results, where infiltration of macrophages and triggered M1 polarization, as well as the release of a large number of inflammatory cytokines (such as IL-1β and TNF-α), were observed in both *in vivo* and *in vitro* models under low shear stress (LSS) conditions (Wei et al., 2024). Specifically, the wall shear stress (WSS) conditions used in these *in vitro* models were 12 dyne/cm^2^ and 5 dyne/cm^2^. Similar results were observed in another *in vitro* experiment, where macrophages exposed to 12 dyne/cm^2^ unidirectional circulation showed significantly elevated M1 markers after 24 h under shear stress conditions (Son et al., 2023). We believe that future experiments should incorporate a wider range of shear stress gradients to help us gain a more comprehensive understanding of how shear stress affects macrophage phenotypic transformation. Additionally, some experiments have used immortalized cell lines, which may, to some extent, affect the reliability of the experimental conclusions.

#### 4.3.3 The role of macrophage-mediated immune cell crosstalk mechanisms in liver immune homeostasis

Liver immune homeostasis is crucial for protecting the liver and preventing long-term chronic inflammatory damage. Macrophages, dendritic cells, and T cells all participate in antigen presentation. However, to prevent abnormal immune activation in response to harmless antigens, these cells remain in a state of immune tolerance and work together to maintain liver immune homeostasis (Méndez-Sánchez et al., 2021). Recent studies have shown that there is a complex crosstalk mechanism between macrophages and other immune cells, including dendritic cells, monocytes, and T cells, and the synergistic actions of these immune cell populations collectively influence liver immune tolerance.

Under normal conditions, hepatic dendritic cells (HDCs) remain in a quiescent state and induce both innate immune tolerance and adaptive T cell tolerance (Ibrahim et al., 2012). HDCs actively contribute to immune suppression by secreting the cytokine IL-10, which inhibits T cell proliferation and induces their deletion, thereby promoting immune tolerance (Méndez-Sánchez et al., 2021; Heymann and Tacke, 2016). IL-10 has significant anti-inflammatory effects, suppressing immune cell activation while neutralizing the activity of pro-inflammatory cytokines (Pratim Das and Medhi, 2023). Another major source of IL-10 under homeostatic conditions is KCs, which can also respond to lipopolysaccharide (LPS) stimulation and secrete IL-10 (Knolle et al., 1995). In the suppression of T cell activation, KCs play a synergistic role. KCs can express the programmed cell death ligand 1 (PD-L1), which inhibits T cell activation (Doherty, 2016). Additionally, KCs suppress T cell activation from other antigen-presenting cells without the need for immunosuppressive cytokines such as IL-10, TGF-β, or nitric oxide (NO) (You et al., 2008; Zheng and Tian, 2019). An *in vitro* coculture study demonstrated that KCs isolated from the livers of naïve mice can suppress dendritic cell (DC)-induced T cell activation by releasing prostaglandin E2 (PGE2) and 15d-PGJ2 (Yan et al., 2010). Both HDCs and KCs can also inhibit T cell proliferation by expressing indoleamine 2,3-dioxygenase (IDO), which is essential for maintaining liver homeostasis (Frumento et al., 2002; Jürgens et al., 2009). IDO is known to exert antimicrobial defense effects by depleting tryptophan (Taylor and Feng, 1991).

#### 4.3.4 A new immunomodulatory strategy targeting macrophage phenotypic transformation to drive fibrosis resolution

Traditional concepts suggest that liver fibrosis is reversible, and the liver’s extensive regenerative capacity makes fibrosis resolution possible (Caligiuri et al., 2021). Macrophages play a crucial role as cellular regulators at various stages of liver fibrosis, participating in chronic inflammation, fibrosis, and resolution through multiple pathways. The complex mechanisms underlying their involvement are primarily related to their phenotypic transformation.

During fibrosis resolution, Ly6C^lo^-marked M2 macrophages dominate. In the early stages of liver injury, Ly6C^lo^ cells promote inflammation resolution and alleviate fibrosis, (Liaskou et al., 2013), playing a key role in clearing cell debris and phagocytosing neutrophils. Once the inflammatory response is reduced, pro-inflammatory M1 macrophages can transition to an inflammation-resolving M2 macrophage phenotype, (Ramachandran et al., 2012), with increased expression of anti-inflammatory genes and metalloproteinases, (Cheng et al., 2021), which are essential for microenvironment remodeling. Additionally, resident macrophages can induce HSCs apoptosis via caspase nine activation, mitigating fibrosis (Fischer et al., 2002). Given the role of liver macrophage subsets in driving fibrosis resolution, inducing their transition from a pro-inflammatory to a regenerative phenotype will favor liver fibrosis resolution. This requires precise immunomodulatory strategies. Various drug delivery systems (liposomes, solid lipids, polymers, or nanoparticles), utilizing the phagocytic and clearing functions of macrophages, can alter targeting properties through intrinsic modifications, offering potential for macrophage-specific therapies (Ergen et al., 2017; Bygd et al., 2017). Among these, liposomes are the most extensively studied and stable carriers, showing significant accumulation in the liver. Liposomes loaded with dexamethasone can deplete monocyte-derived macrophages (MoMFs) and promote macrophage polarization towards the M2 phenotype. Notably, liposome-encapsulated dexamethasone is more effective than free dexamethasone in clearing T cells in the blood and liver, promoting anti-inflammatory polarization of liver macrophages, and significantly alleviating liver injury and fibrosis (Bartneck et al., 2015). Dejun Yang and colleagues developed cationic liposomes encapsulating oleanolic acid (OA) and synthesized cerium oxide nanomaterials using hydroxyapatite (HA) as a template. HA specifically binds to the CD44 receptor, which is overexpressed on the surface of macrophages and HSCs, enabling precise drug delivery to these cells. OA exhibits anti-inflammatory, antioxidant, and hepatoprotective bioactivities. The study showed that these liposomes could alleviate the inflammatory microenvironment in liver fibrosis models and promote macrophage polarization from the pro-inflammatory M1 phenotype to the anti-inflammatory M2 phenotype (Yang et al., 2024).

Additionally, nanoparticle-encapsulated herbal monomers are an effective approach to modulate macrophage phenotypes in Traditional Chinese Medicine (TCM). Administration of turmeric encapsulated in CD44-targeting hyaluronate–polylactide (HA-PLA) nanoparticles (NPs) can promote the transition of M1 macrophages to the M2 phenotype, reduce iNOS levels, and upregulate Arg-1 expression (Farajzadeh et al., 2018). Administering quercetin encapsulated in collagenase-modified melanin nanoparticles (MNPs) can reduce excessive collagen deposition. It also exerts a synergistic anti-inflammatory and reactive oxygen species (ROS) scavenging effect of both quercetin and melanin, regulating macrophage M1-M2 polarization and inhibiting the release of pro-inflammatory cytokines (Li et al., 2025). These studies demonstrate the therapeutic potential of DDS (drug delivery systems) for precise immune modulation targeting macrophages to reverse liver fibrosis. However, most of the research is still in the preclinical stage.

## 5 Summary and outlook

This study combines bibliometric analysis with a review of research hotspots related to the pathological mechanisms of macrophages in liver fibrosis, offering certain advantages compared to previous studies that solely relied on bibliometric analysis or narrative reviews. To our knowledge, this is the first bibliometric study focusing on the relationship between macrophages and liver fibrosis. However, this study also has some limitations. For example, only one database (WoSCC) was searched, it only includes English-language literature, and it only considers literature published between 2013 and 2023. These limitations may introduce potential bias. In addition, the visualization results of this study only show the overall research structure and development of the field. In subsequent studies we will conduct deep relational analyses and try to further explore the complexity of relationships in the field by combining temporal cooperative evolution, causal mining of urgent themes.

Although our findings suggest that the literature on macrophages’ involvement in the pathological mechanisms of liver fibrosis has rapidly increased, several significant challenges and unresolved issues remain. First, a more complex and refined classification of macrophage subsets needs to be clarified, including information on the time and location of macrophage subset accumulation in liver tissue. These valuable details will help us better understand the complex processes in which macrophages participate in liver fibrosis. Currently, single-cell sequencing and spatial transcriptomics have yielded some findings, but further and deeper research is necessary. Second, mechanistic immunology may represent an emerging research direction to explore the crosstalk between macrophages and hepatic stellate cells. However, the understanding of how extracellular matrix stiffness regulates macrophage phenotypic transformation and affects hepatic stellate cell activation is limited and controversial. We believe it is essential to set up more groups with varying extracellular matrix stiffness and shear stress conditions to explore these patterns. Furthermore, research on targeting macrophage phenotypic transformation to drive the regression of liver fibrosis is mostly still in the preclinical stage. Based on our findings, these areas represent key directions for future research.
